# Tracking of physical activity, fitness, body composition and diet from adolescence to young adulthood: The Young Hearts Project, Northern Ireland

**DOI:** 10.1186/1479-5868-1-14

**Published:** 2004-10-05

**Authors:** Colin Boreham, Paula J Robson, Alison M Gallagher, Gordon W Cran, J Maurice Savage, Liam J Murray

**Affiliations:** 1 School of Applied Medical Sciences and Sports Studies, University of Ulster, Jordanstown, Northern Ireland, BT37 0QB, United Kingdom; 2 Northern Ireland Centre for Food and Health (NICHE), University of Ulster, Coleraine, Northern Ireland, BT52 1SA, United Kingdom; 3 Department of Epidemiology and Public Health, Queen's University of Belfast, Belfast, Northern Ireland, BT12 6BJ, United Kingdom; 4 Department of Child Health, Queen's University of Belfast, Belfast, Northern Ireland, BT12 6BJ, United Kingdom

## Abstract

**Background:**

The assumption that lifestyles formed early in life track into adulthood has been used to justify the targeting of health promotion programmes towards children and adolescents. The aim of the current study was to use data from the Northern Ireland Young Hearts Project to ascertain the extent of tracking, between adolescence and young adulthood, of physical activity, aerobic fitness, selected anthropometric variables, and diet.

**Methods:**

Males (*n *245) and females (*n *231) were assessed at age 15 y, and again in young adulthood [mean (SD) age 22 (1.6) y]. At both timepoints, height, weight and skinfold thicknesses were measured, and physical activity and diet were assessed by questionnaire and diet history method respectively. At 15y, fitness was assessed using the 20 metre shuttle run, while at young adulthood, the PWC170 cycle ergometer test was used. For each measurement made at 15y, subjects were ranked into 'low' (L1; lowest 25%), 'medium' (M1; middle 50%) or 'high' (H1; highest 25%) categories. At young adulthood, similar categories (L2, M2, H2) were created. The extent of tracking of each variable over time was calculated using 3 × 3 matrices constructed using these two sets of categories, and summarised using kappa (κ) statistics.

**Results:**

Tracking of diet and fitness was poor (κ ≤ 0.20) in both sexes, indicating substantial drift of subjects between the low, medium and high categories over time. The tracking of physical activity in males was fair (κ 0.202), but was poor in females (κ 0.021). In contrast, anthropometric variables such as weight, body mass index and sum of skinfolds tracked more strongly in females (κ 0.540, κ 0.307, κ 0.357 respectively) than in males (κ 0.337, κ 0.199, κ 0.216 respectively).

**Conclusions:**

The poor tracking of fitness and diet in both sexes, and physical activity in females, suggests that these aspects of adolescent lifestyle are unlikely to be predictive of behaviours in young adulthood. In contrast, the fair to moderate tracking of anthropometric variables, particularly in females, suggests that attempts to reduce the ever increasing incidence of overweight and obesity in adults, should probably begin in earlier life.

## Background

Numerous epidemiological studies in adults have identified environmental and physiological risk factors that are associated with increased risk for cardiovascular disease (CVD). Among the many that have been identified [1], the major modifiable risk factors include physical inactivity, poor cardiorespiratory fitness, excess adiposity or obesity, and inappropriate dietary habits.

Although the clinically relevant effects of CVD are often not manifest until middle age or later, it is now generally well accepted that the disease is likely to have its antecedents in childhood [2-4]. In addition, children and adolescents have been shown to exhibit many of the potentially modifiable CVD risk factors that have been identified in adults. For example, of the 1015 adolescents aged 12y and 15y who participated in the first phase of the Northern Ireland Young Hearts Project, 18–34% were considered to have excess body fat, 24–29% had low physical activity levels, and 26–34% had poor cardiorespiratory fitness. Furthermore, mean total fat intakes were higher than desirable [5]. Similarly, in West Virginia, of the 5,887 school children who participated in the school-based Coronary Artery Risk Detection in Appalachian Communities (CARDIAC) Project, almost 43 percent were considered to be overweight, over a quarter were obese, and the high rate of obesity was positively associated with the prevalence of other CVD risk factors [6]. The conclusions drawn from these studies and others [7-9] have been largely unanimous: minimising the risk of morbidity or premature mortality associated with CVD in adulthood, should begin in childhood or adolescence.

However, if health promotion interventions in younger life are to have any hope of success, it must be assumed that physiological and behavioural risk factors exhibited in early life track into adulthood. Tracking has been defined as the maintenance of relative position in rank of behaviour over time, such that subjects who rank highly for unfavourable risk profiles at a young age are likely to maintain their ranks through into adulthood [10,11].

The aim of the current study was to use data from the Northern Ireland Young Hearts Project to ascertain the extent of tracking, between adolescence and young adulthood, of selected modifiable risk factors for CVD. Specifically, we investigated the tracking of physical activity, aerobic fitness, selected anthropometric variables, and diet.

## Methods

### Subjects

The Young Hearts Project (YH) is an ongoing longitudinal study evaluating the prevalence of CVD risk factors in young people living in Northern Ireland. Sampling procedures and methods used in the first two, school-based screening phases of the YH study have been described fully elsewhere [5]. Briefly, the initial screening (YH1), conducted in 1989/1990, surveyed 1015 adolescents (12-year old boys, *n *251;12-year old girls, *n *258; 15-year old boys, *n *252; and 15-year old girls, *n *254) randomly selected from post-primary schools. At that time, the resulting cohort represented a 2% sample of each of the two age populations in Northern Ireland. In 1992/93, a follow up study (YH2) was undertaken, in which subjects from the original 12 year old cohort were reassessed using procedures identical to those used in YH1. The response rate in the follow-up study was 90%. Between October 1997 and October 1999, all YH1 subjects were invited to participate in the third, hospital-based screening phase (YH3), and a 48.2% response rate was achieved. Reasons for the low response rate in YH3 have been described in full elsewhere [12]. Briefly, non-attenders reported that they were 'too busy', 'living outside Northern Ireland', 'busy with new job', 'couldn't be bothered' or 'didn't feel that the study was relevant to them'. As described by [12] and [13], attempts were made to determine the representativeness of the YH3 cohort by comparing the baseline YH1 data for those who participated in YH3, with the data obtained for those who declined to participate. YH3 participants tended to be from families with higher socio-economic status, and had lower BMI at baseline (YH1) than non-participants. Furthermore, males who declined to attend for screening at YH3, were fatter and reported a greater saturated fat intake at YH1 than YH3 male participants.

The analyses reported in the current paper are restricted to males (*n *245) and females (*n *231) for whom there were complete data sets at age 15y (either from YH1 or YH2) and at young adulthood [mean (SD) age 22.0 (1.6)y]. Ethical approval for each phase of the study was obtained from the Medical Research Ethical Committee of The Queen's University of Belfast, and written informed consent was obtained from all subjects prior to participation.

### Anthropometry

Each subject's height, weight and skinfold thicknesses were measured at all study timepoints. Standing height was measured to the nearest millimetre using a Harpenden portable stadiometer (Holtain, UK), and body weight was measured to the nearest 0.1 kg using an electronic balance (Seca, Germany; 200 kg × 0.1 kg). For both measurements, subjects wore light indoor clothing and no shoes. Body mass index (BMI) was then calculated as weight (kg)/ [height (m)]^2^. Skinfold thicknesses were measured to the nearest millimetre using Harpenden callipers at four sites (biceps, triceps, subscapular, suprailiac). Two measurements were taken at each site and the average was recorded. The sum of the four skinfolds thicknesses was then calculated for each subject.

### Dietary intake

At all study timepoints, dietary data were obtained using the diet history method [14]. This consisted of a detailed, open-ended one-to-one interview, the purpose being to ascertain the habitual weekly food intake of each subject. The diet history method was used for two reasons. Firstly, in subjects aged 15y, the diet history has been shown to provide more valid estimates of energy intake at the group level than weighed records [15]. Secondly, given that a complete diet history can be obtained from a subject in approximately one hour, it was the most feasible and cost-effective method for obtaining detailed dietary information from the YH1 and YH2 school-based cohorts. The method was used again in YH3 in order to maintain continuity. Reported energy and macronutrient intakes were calculated using computerised databases based on UK food composition tables as previously described [16,12]

### Physical activity

At age 15y, habitual physical activity was assessed by self-report questionnaire, and scored according to the method of [17]. This method assessed the extent of daily participation in activities that were based around a typical school day. Each activity was assigned a score from 1–100, based on its frequency, intensity and duration.

As the school-based questionnaire was not relevant to the young adult subjects, a modification [18] of the Baecke questionnaire was used in YH3 to quantify habitual work activity, sports activity and non-sports leisure activity. For each of the three activity components, scores based on a five-point Likert scale were calculated and summed, giving total possible scores ranging from 3–15.

### Aerobic fitness

Aerobic fitness at age 15y was assessed by the 20 metre shuttle test (20MST). In order to estimate maximal aerobic capacity, or VO_2_max (ml/kg/min), the number of laps completed by each subject in this maximal endurance test was entered into a sex-specific regression equation, based on data obtained in the Northern Ireland Fitness Survey [17].

As it was not feasible to conduct the 20MST at young adulthood (due to a lack of space in the hospital setting), VO_2_max was assessed using the Physical Work Capacity at a heart rate of 170 beats per minute (PWC170) cycle ergometer test [19]. PWC170 was calculated as the workload corresponding to a heart rate of 170 bpm, and expressed per kg body weight. The volume of oxygen consumed and the heart rate were monitored throughout the test (Quinton Metabolic Cart, Quinton, USA). For each subject, a straight line was fitted to three pairs of data (heart rate in bpm, VO_2 _in ml/kg/min), and this was used to estimate VO_2_max at the age-adjusted maximum heart rate [12].

### Statistical analyses

All data were analysed using SPSS version 11.0.1 (SPSS Inc, Chicago, USA). Means and standard deviations were used to summarise the data for physical characteristics, aerobic fitness, physical activity scores and energy and macronutrient intakes of males and females at age 15y and young adulthood.

**Figure 1:**
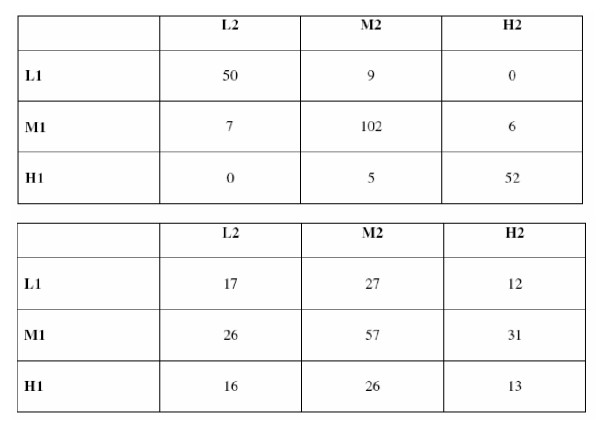
Examples of 3 × 3 tracking matrices constructed for the calculation of κ values for (a) height in females, and (b) physical activity scores in females.(a) Height in females (κ 0.813) (b) Physical activity score in females (κ 0.021) L1 and L2 represent lowest 25% of the cohort at baseline and follow-up respectively; M1 and M2 represent middle 50% of the cohort at baseline and follow-up respectively; H1 and H2 represent the highest 25% of the cohort at baseline and follow-up respectively. The entry in a specific cell indicates the number of subjects belonging to the corresponding classes at baseline and at follow-up.

Tracking of each of these variables over time was assessed by determining the extent to which subjects who were placed into low, medium and high categories at age 15y, maintained their ranking in young adulthood. Owing to the fact that different techniques were used to measure physical activity and aerobic fitness at each timepoint, a method based on ranks, rather than actual measurements, was employed for assessing the tracking of these variables. Tracking of the other variables was also assessed using the rank based method because of its relative simplicity, and its ability to show the numbers of subjects making the transition between low, medium and high categories [20]. For example, in order to study the tracking of physical activity in females from age 15y to young adulthood, the group of 225 girls aged 15y was divided into three classes by physical activity score: lowest 25% (L1); middle 50% (M1); highest 25% (H1). Rather than using pre-determined fixed values, each class was defined by the first and third empirical quartiles. In young adulthood, the female group was divided into three similar classes; L2, M2 and H2. Using these two sets of classifications, a 3 × 3 tracking matrix was constructed; the entry in a specific cell being the number of subjects belonging to the corresponding classes at age 15y and at young adulthood (see Figure 1 for examples). This approach provides a broad picture of the relative changes in a particular variable over time, such that a matrix with relatively small off-diagonal elements provides evidence of 'good' tracking. For the purposes of this study, the degree of tracking was summarised by a weighted kappa (κ) value, and interpreted according to [21] as follows: κ ≤ 0.20, poor tracking; κ 0.21–0.40, fair; κ 0.41–0.60, moderate; κ 0.61–0.8, good; κ 0.81–1.0, very good. This procedure was undertaken separately for males and females to assess the tracking, between age 15y and young adulthood, of energy and macronutrient intakes, height, weight, BMI, skinfold thicknesses, aerobic fitness and physical activity scores.

## Results

The physical characteristics, aerobic fitness and physical activity levels of the Northern Ireland Young Hearts cohort at age 15y and at follow-up (young adulthood) are summarised in Table 1. At young adulthood, weight, height, BMI and skinfold thicknesses were significantly greater than at age 15y in males and females. In both sexes, VO_2_max assessed at young adulthood was significantly lower than at age 15y. At age 15y, males and females were 4.8% and 8.4% heavier than the British age-specific reference population, while at young adulthood, they were 7.9% and 9.9% heavier respectively. Details of the reference populations are described in Annex 1 of the '*Dietary Reference Values for Food Energy and Nutrients for the United Kingdom*' [22].

**Table 1:** Physical characteristics, fitness and physical activity levels at age 15y, and at young adulthood (mean age 22.0y).

	Males						Females					
	Baseline			Follow-up			Baseline			Follow-up		

	n	Mean	SD	n	Mean	SD	n	Mean	SD	n	Mean	SD

Weight (kg)	245	59.2	8.9	245	75.5***	11.5	231	56.9	9.1	231	64.3***	11.7
Height (m)	245	1.70	0.07	245	1.78***	0.07	231	1.62	0.06	231	1.64***	0.06
Body mass index (kgm^-2^)	245	20.4	2.4	245	23.8***	3.1	231	21.7	3.2	231	23.8***	4.1
Biceps skinfold (mm)	245	4.73	2.23	244	5.54***	3.42	231	8.00	2.92	230	9.57***	5.27
Triceps skinfold (mm)	245	9.21	4.59	244	10.28**	5.41	231	15.97	4.49	230	17.84***	5.94
Subscapular skinfold (mm)	245	7.75	3.75	244	12.94***	5.22	231	11.57	4.77	230	15.14***	6.17
Suprailiac skinfold (mm)	245	10.30	6.58	244	15.87***	7.43	231	14.08	5.73	230	16.01***	6.98
Sum of skinfolds (mm)	245	32.00	16.07	244	44.63***	18.76	231	49.61	15.62	230	58.56***	20.76
VO_2_max^a^	241	52.07	5.96	225	38.93***	8.70	228	41.05	5.48	212	26.90***	5.43
Physical activity score^b^	242	28.27	14.44	243	7.95	1.38	227	17.71	12.59	229	7.40	1.20

^a ^VO_2_max at age 15y was derived from the number of 20 metre shuttle run laps completed by each subject. At follow-up, VO_2_max was extrapolated from the results of a cycle ergometer test (Physical Work Capacity at a heart rate of 170 bpm; PWC170).

^b ^Physical activity scores at age 15y were calculated according to the method of Riddoch *et al *(1991). At follow-up, a modification (Pereira *et al*, 1997) of the Baecke questionnaire was used. At age 15y, the maximum possible activity score was 100, while at follow-up, scores could range from 3–15.

For each sex, differences between variables measured at age 15y and young adulthood were assessed using paired t-tests. *** *P *< 0.001 ** *P *< 0.01.

The energy and macronutrient intakes reported by the Young Hearts cohort at age 15y, and at follow-up, are presented in Table 2. At young adulthood, the males reported significantly lower intakes of energy (MJ/d; *P *0.04), total fat (g/d and % energy; both *P *< 0.001) and total carbohydrate (g/d and % energy; both *P *< 0.001) than at age 15y. In contrast, intakes of protein (g/d and % energy; both *P *< 0.001) reported by males at young adulthood were significantly greater than at age 15y. Similar patterns were observed for females, with the exception that % energy derived from total carbohydrate did not change significantly between age 15y and young adulthood.

**Table 2:** Energy and macronutrient in takes^a ^reported at age 15y, and at young adulthood (mean age 22.0y).

	Males (*n *245)				Females (*n *231)			
	Baseline		Follow-up		Baseline		Follow-up	

	Mean	SD	Mean	SD	Mean	SD	Mean	SD

Energy (MJ/d)	13.5	3.2	13.0*	3.5	9.4	2.6	8.3***	2.4
Protein (g/d)	95.0	25.6	101.2**	27.9	63.9	18.6	68.5**	21.4
% energy from protein	12.0	1.9	12.6***	2.3	11.6	2.1	13.4***	2.9
Total fat (g/d)	137.1	38.5	113.2***	38.3	96.8	30.3	73.3***	25.0
% energy from fat	37.4	4.3	32.1***	5.5	37.7	4.3	32.5***	6.0
Carbohydrate (g/d)	411.6	100.3	368.6***	110.5	289.6	82.2	253.6***	87.1
% energy from carbohydrate	48.8	4.6	45.7***	7.2	49.2	4.9	49.1^NS^	6.5

^a ^At both timepoints, energy and macronutrient intakes were assessed using the diet history method.

For each sex, differences between variables measured at age 15y and young adulthood were assessed using paired t-tests. *** *P *< 0.001 ** *P *< 0.01 NS not significant

Table 3 summarises the extent of tracking of physical characteristics, fitness and physical activity levels between age 15y and young adulthood, in males and females. In males, tracking of height was moderate, while in females, it was very good. Figure 1(a), which illustrates the 3 × 3 matrix constructed for the tracking of height in females between age 15y and young adulthood, demonstrates that there was very little drift of subjects between low, medium and high categories over time; hence the relatively high κ value. In both sexes, tracking of BMI was moderate. In males, the tracking of weight, four skinfold thicknesses (biceps, triceps, subscapular, suprailiac) and sum of skinfolds was fair (κ 0.21–0.40) between age 15y and young adulthood. The magnitude of the κ values obtained for biceps and subscapular skinfold thicknesses in females were also greater than in males, while triceps and suprailiac skinfold thicknesses tracked to a similar extent in both sexes. In males, the tracking of aerobic fitness (VO_2_max) was poor, but was greater than the κ value obtained for the females (κ 0.150 *vs *κ 0.076). A similar pattern was observed for physical activity scores (κ 0.202 *vs *κ 0.021).

**Table 3:** Tracking of physical characteristics, fitness and physical activity levels between age 15y and young adulthood (mean age 22.0y)

	Males			Females		
	n	κ	P	n	κ	P

Weight (kg)	245	0.337	<0.0001	231	0.540	<0.0001
Height (m)	245	0.444	<0.0001	231	0.813	<0.0001
Body mass index (kgm^-2^)	245	0.422	<0.0001	231	0.452	<0.0001
Biceps skinfold (mm)	244	0.224	<0.0001	230	0.403	<0.0001
Triceps skinfold (mm)	244	0.292	<0.0001	230	0.287	<0.0001
Subscapular skinfold (mm)	244	0.274	<0.0001	230	0.371	<0.0001
Suprailiac skinfold (mm)	244	0.223	<0.0001	230	0.202	<0.0001
Sum of skinfolds (mm)	244	0.216	<0.0001	230	0.357	<0.0001
VO_2_max^a^	222	0.150	<0.0001	209	0.076	0.128
Physical activity score^b^	240	0.202	<0.0001	225	0.021	0.669

^a ^VO_2_max at age 15y was derived from the number of 20 metre shuttle run laps completed by each subject. At follow-up, VO_2_max was extrapolated from the results of a cycle ergometer test (Physical Work Capacity at a heart rate of 170 bpm; PWC170).

^b ^Physical activity scores at age 15y were calculated according to the method of Riddoch *et al *(1991). At follow-up, a modification (Pereira *et al*, 1997) of the Baecke questionnaire was used.

κ indicates extent of tracking, and can be interpreted as follows: κ < 0.20, poor tracking; κ 0.21–0.40, fair; κ 0.41–0.60, moderate; κ 0.61–0.8, good; κ 0.81–1.0, very good (Altman, 1991).

The extent of tracking of energy and macronutrient intakes reported at age 15y and at young adulthood is presented in Table 4. In males, the κ values for energy, protein, total fat and total carbohydrate were poor, ranging from 0.019 (% energy from protein) to 0.169 (energy). Similarly, κ values observed in the females ranged from 0.051 (% energy from fat) to 0.202 (protein).

**Table 4:** Tracking of energy and macronutrient intakes^a ^between age 15y and young adulthood (mean age 22.0y).

	Males			Females		
	n	κ	P	n	κ	P

Energy (MJ/d)	245	0.169	<0.0001	231	0.154	0.001
Protein (g/d)	245	0.169	<0.0001	231	0.202	<0.0001
% energy from protein	245	0.019	0.683	231	0.098	0.039
Total fat (g/d)	245	0.117	0.011	231	0.152	0.001
% energy from fat	245	0.143	0.002	231	0.051	0.282
Carbohydrate (g/d)	245	0.114	0.013	231	0.120	0.011
% energy from carbohydrate	245	0.117	0.011	231	0.063	0.182

^a ^At both timepoints, energy and macronutrient intakes were assessed using the diet history method.

κ indicates extent of tracking, and can be interpreted as follows: κ < 0.20, poor tracking; κ 0.21–0.40, fair; κ 0.41–0.60, moderate; κ 0.61–0.8, good; κ 0.81–1.0, very good (Altman, 1991).

## Discussion

The current paper describes the extent of tracking for a range of behavioural and biological risk factors for CVD, between adolescence (15y) and young adulthood (22y) in 245 males and 231 females from Northern Ireland.

In relation to nutrient intakes, the poor tracking between 15 and 22 years revealed in this study reflects previous findings in this cohort between 12 and 15 years [20]. This suggests that individual dietary patterns exhibited at 15 years are unlikely to be predictive of dietary intakes at young adulthood. Intuitively, this lack of tracking is to be expected, as the transition from adolescence to adulthood is characterised by considerable physical, cognitive and psychosocial change. To date, however, there has been little evidence for tracking or otherwise of diet in this age group. Reasonably good dietary tracking has been reported for younger pre-school children [23], but this is not surprising given the high degree of control over diet exerted by parents in this age group. Reasonably high tracking coefficients for diet in the Amsterdam Growth and Health Longitudinal Survey were reported by Kemper et al. [24], but these were between the ages of 13 and 32 years. Although it is difficult to draw direct comparisons between studies due to differences in methodologies, one possibility for the poor tracking reported in the present study might be a particularly high degree of mis-reporting of intake in 15 year-old adolescents. This has been noted previously in relation to this cohort, particularly in relation to 'under-reporting' in 15 year-old females [20]. It is also possible that the low κ values obtained for the dietary intakes in the present study indicate that adolescence is, indeed, associated with rapidly changing and erratic patterns of nutrient intake. Adolescents take increasing control of what, when and where they eat and typically consume a greater proportion of their total intake outside the home. Concerns about changing body shape and adiposity may also prompt sudden changes in eating behaviour.

While adolescence is widely regarded to be a time of transition, it could also be argued that young adulthood is an equally important time of change, especially with regard to dietary habits. This is a time when people in this age group are likely to move out of home, go to university, start a family or to encounter other environmental or psychosocial factors that influence food intakes. Thus it is possible that the poor maintenance of ranks that we have observed in this study has arisen simply because we attempted to assess tracking between two very unstable periods of time in the life-cycle.

It is also possible that at least part of the explanation for poor dietary tracking in a cohort of this nature lies in the unsuitability of the diet history method for this purpose. Although the diet history method has shown good validity at the group level in adolescents, it is prone to significant problems of precision at the individual level [15]. Moreover, as it assesses perception and memory of usual diet and is susceptible to socially desirable responding [25], it is possible that changes in memory and motivation over time may contribute to poor tracking. Finally, it is entirely feasible that diet simply does not track well between two time points several years apart. Certainly, the data presented in this study suggest that individual dietary patterns reported at 15 years are unlikely to be predictive of energy and nutrient intakes reported at 22 years. It is clear, therefore, that individual subjects cannot be targeted for long-term dietary intervention based solely on data obtained at 15 years of age.

Both physical fitness and physical activity are now accepted as independent risk factors for several chronic diseases. The identification of low levels of fitness and/or activity at an early stage in the lifecourse might, therefore, enable early remedial strategies, provided that a degree of tracking for these risk factors is demonstrable. The results of the present study fail to provide such evidence, with poor tracking being demonstrated for fitness in both sexes, and poor tracking in females and only moderate levels of tracking in males for activity. By and large, these results are in keeping with the handful of other studies which have examined tracking of fitness and activity in this age group [26,27]. While the mechanisms responsible for this lack of stability between adolescence and early adulthood remain obscure, it is tempting to speculate that similar influences are responsible for the poor tracking of both diet and physical activity/fitness. For example, while much activity during adolescence is organised and school-based, by the time the individual reaches early adulthood, activity is likely to be more a matter of choice. In this respect, it is interesting to note that studies of the tracking of physical activity/fitness in both younger [28] and older [29,30] age groups than that of the present study, show generally higher levels of tracking between time points.

In contrast to diet and physical activity/fitness, anthropometric variables relating to body weight and adiposity showed stronger and consistent tracking, particularly in females. Good tracking of BMI from adolescence to young adulthood has been noted in previous studies [31,24,33], with stronger tracking for females also highlighted. The results of the present study thus confirm the potential utility of identifying adolescents at the age of 15 years who are at risk of persistent obesity, and targeting such adolescents with appropriate long-term lifestyle advice. Our results for diet, physical activity and fitness, however imply greater instability from adolescence to young adulthood, and consequently the need for shorter-term, ameliorative strategies based on regular monitoring of these behaviours and attributes.

## Competing Interests

The authors declare that they have no competing interests.

## Authors' contributions

CB conceived of the study, participated in its design and co-ordination and co-drafted the manuscript. PR supervised the collection of dietary data and co-drafted the manuscript. AG and GC carried out the statistical analysis. LM participated in the design and co-ordination of the project. JS conceived of the study and participated in its design and co-ordination. All authors read and approved the final manuscript.

## References

[B1] Hopkins PN, Williams RR (1986). Identification and relative weight of cardiovascular risk factors. Cardiol Clin.

[B2] Eskola O (1948). On the occurrence of arteriosclerosis in Finland. Duodecim.

[B3] Enos WF, Holmes RH, Beyer J (1953). Coronary disease among United States soldiers killed in action in Korea: preliminary report. J Am Med Assoc.

[B4] McGill HC, McMahan CA, Herderick EE, Zieske AW, Malcom GT, Tracy RE, Strong JP (2002). Pathobiological Determinants of Atherosclerosis in Youth (PDAY) Research Group. Obesity accelerates the progression of coronary atherosclerosis in young men. Circulation.

[B5] Boreham C, Savage JM, Primrose D, Cran G, Strain J (1993). Coronary risk factors in schoolchildren. Arch Dis Child.

[B6] Muratova VN, Demerath EW, Spangler E, Ogershok P, Elliott E, Minor VE, Neal WA (2002). The relation of obesity to cardiovascular risk factors among children: the CARDIAC project. W V Med J.

[B7] Harsha DW, Smoak CG, Nicklas TA, Webber LS, Berenson GS (1987). Cardiovascular risk factors from birth to 7 years of age: the Bogalusa Heart Study. Tracking of body composition variables. Pediatrics.

[B8] Gliksman MD, Dwyer T, Wlodarczyk J (1990). Differences in modifiable cardiovascular disease risk factors in Australian schoolchildren: the results of a nationwide survey. Prev Med.

[B9] Berenson GS, Srinavasan S, Webber LS, Nicklas TA, Hunter SM, Harsha DW (1991). Cardiovascular risk in early life: The Bogalusa Heart Study.

[B10] Kelder SH, Perry CL, Klepp K-I, Lytle LL (1994). Longitudinal tracking of adolescent smoking, physical activity and food choice behaviours. Am J Public Health.

[B11] Twisk JWR, Kemper HCG, van Mechelen W, Post GB (1997). Tracking of risk factors for coronary heart disease over a 14-year period: a comparison between lifestyle and biologic risk factors with data from the Amsterdam Growth and Health Study. Am J Epidemiol.

[B12] Gallagher AM, Savage JM, Murray LJ, Davey Smith G, Young IS, Robson PJ, Neville CE, Cran G, Strain JJ, Boreham CA (2002). A longitudinal study through adolescence to adulthood: the Young Hearts Project, Northern Ireland. Public Health.

[B13] van Lenthe FJ, Boreham CA, Twisk JWR, Savage MJ, Murray L, Davey Smith G (2001). What determines drop-out in prospective studies of coronary heart disease risk factors between youth and young adulthood: the Young Hearts Study. J Epidemiol Comm Health.

[B14] van Staveren WA, de Boer JD, Burema J (1985). Validity and reproducibility of a dietary history method estimating the usual food intake during one month. Am J Clin Nutr.

[B15] Livingstone MBE, Prentice AM, Coward WA, Strain JJ, Black AE, Davies PS, Stewart CM, McKenna PG, Whitehead RG (1992). Validation of estimates of energy intake by weighed dietary record and diet history in children and adolescents. Am J Clin Nutr.

[B16] Strain JJ, Robson PJ, Livingstone MBE, Primrose ED, Savage JM, Cran GW, Boreham CA (1994). Estimates of food and macronutrient intake in a random sample of Northern Ireland adolescents. Br J Nutr.

[B17] Riddoch C, Savage JM, Murphy N, Cran GW, Boreham C (1991). Long term health implications of fitness and physical activity patterns. Arch Dis Child.

[B18] Pereira MA, Fitzgerald SJ, Gregg EW, Joswiak ML, Ryan WJ, Suminski RR, Utter AC, Zmuda JM (1997). Baecke questionnaire of habitual activity. Med Sci Sports Exerc,.

[B19] Council of Europe. Committee of experts on sports research – EUROFIT (1993). Handbook for the EUROFIT tests on physical fitness.

[B20] Robson PJ, Gallagher AM, Livingstone MBE, Cran GW, Strain JJ, Savage JM, Boreham CAG (2000). Tracking of nutrient intakes in adolescence: the experiences of the Young Hearts Project, Northern Ireland. Br J Nutr.

[B21] Altman DG (1991). Practical Studies for Medical Research.

[B22] Department of Health (1991). Dietary reference values for food energy and nutrients for the United Kingdom.

[B23] Stein AD, Shea S, Basch CE, Contento IR, Zybert P (1991). Variability and tracking of nutrient intakes of preschool children based on multiple administrations of the 24-hour dietary recall. Am J Epidemiol.

[B24] Kemper HC, Post GB, Twisk J, van Mechelen W (1999). Lifestyle and obesity in adolescence and young adulthood results from the Amsterdam Growth and Health Longitudinal Survey (AGAHLS). Int J Obes Relat Metab Disord.

[B25] Livingstone MB, Robson P (2000). Measurement of dietary intake in children. Nut Proc Soc.

[B26] Telama R, Leskinen E, Yang X (1996). Stability of habitual physical activity and sports participation: a longitudinal tracking study. Scand J Med Sci Sports.

[B27] Cambell PT, Katzmarzyk PT, Malina RM, Rao DC, Perusse L, Bouchard C (2001). Prediction of physical activity and physical work capacity (PWC 150) in young adulthood from childhood and adolescence with consideration of parental measures. Am J Human Biol.

[B28] Janz K, Dawson J, Mahoney L (2000). Tracking physical fitness and physical activity from childhood to adolescence: The Muscatine Study. Med Sci Sports Exerc.

[B29] Anderssen N, Jacobs D, Sidney S, Bild D, Sternfeld B, Slattery ML, Hannan P (1996). Change and secular trends in physical activity patterns in young adults: a seven-year longitudinal follow-up in the Coronary Artery Risk Development in Young Adults Study (CARDIA). AM J Epidemiol.

[B30] De Bourdeaudhuij I, Sallis J, Vandelanotte C (2002). Tracking and explanation of physical activity in young adults over a seven-year period. Res Q Exerc Sport.

[B31] Guo S, Roche A, Chumlea W, Gardner J, Siervogel R (1994). The predictive value of childhood body mass index values for overweight at age 35 y. Am J Clin Nutr.

[B32] Trudeau F, Shephard RJ, Arsenault F, Laurencelle L (2001). Changes in adiposity and body mass index from late childhood to adult life in the Trois-Rivieres study. Am J Human Biol.

[B33] Margarey A, Daniels L, Boulton T, Cockington R (2003). Predicting obesity in early adulthood from childhood and parental obesity. Int J Obes.

